# Insights into the prevalence and underlying causes of clonal variation through transcriptomic analysis in *Pichia pastoris*

**DOI:** 10.1007/s00253-017-8317-2

**Published:** 2017-05-22

**Authors:** Rochelle Aw, Geraint R Barton, David J. Leak

**Affiliations:** 1 Department of Life Sciences, Imperial College London, London, SW7 2AZ UK; 2 Centre for Synthetic Biology and Innovation, Imperial College London, London, SW7 2AZ UK; 3 Centre for Integrative Systems Biology and Bioinformatics, Imperial College London, London, SW7 2AZ UK; 4 Department of Biology & Biochemistry, University of Bath, Bath, BA2 7AY UK

**Keywords:** *Pichia pastoris/Komagataella phaffii*, Transcriptomic analysis/microarray, Clonal variation, Unfolded protein response, ER-associated degradation, Protein expression

## Abstract

Clonal variation, wherein a range of specific productivities of secreted proteins are observed from supposedly identical transformants, is an accepted aspect of working with *Pichia pastoris.* It means that a significant number of transformants need to be tested to obtain a representative sample, and in commercial protein production, companies regularly screen thousands of transformants to select for the highest secretor. Here, we have undertaken a detailed investigation of this phenomenon by characterising clones transformed with the human serum albumin gene. The titers of nine clones, each containing a single copy of the human serum albumin gene (identified by qPCR), were measured and the clones grouped into three categories, namely, high-, mid- and low-level secretors. Transcriptomic analysis, using microarrays, showed that no regulatory patterns consistently correlated with titer, suggesting that the causes of clonal variation are varied. However, a number of physiological changes appeared to underlie the differences in titer, suggesting there is more than one biochemical signature for a high-secreting strain. An anomalous low-secreting strain displaying high transcript levels that appeared to be nutritionally starved further emphasises the complicated nature of clonal variation.

**Electronic supplementary material:**

The online version of this article (doi:10.1007/s00253-017-8317-2) contains supplementary material, which is available to authorized users.

## Introduction

The phenomenon of clonal variation has been noted in a variety of organisms, most noticeably Chinese hamster ovary (CHO) cells where it was first described in 1977 (Konrad et al. 1977). Supposedly genetically identical clones plated on agar plates showed variation in colony morphology, as well as variation when used for the production of recombinant proteins. Differences were most noticeable in secreted protein titer and highlighted an underlining heterogeneity in CHO cells that had not previous been identified (Dahodwala et al. 2012; O’Callaghan and James 2008; Pilbrough et al. 2009). Variation has often been attributed to differences in gene integration sites, the use of antibiotics for selection or gene copy number (Kim et al. 2001; Zdzienicka et al. 1985). Variation in plant cells, known as somaclonal variation, is predominantly attributed to stress factors, although epigenetic factors such as copy number variation, gene silencing and gene activation have been noted (Bardini et al. 2003; Kaeppler et al. 2000; Kidwell and Osborn 1993).

The methylotrophic yeast *Pichia pastoris*, reclassified as *Komagataella phaffii/pastoris*, was described as showing clonal variation in the mid-1980s (Cregg et al. 1989). Indeed, clonal variation is such an integral feature that for commercial production using *P. pastoris* as an expression host, screening hundreds of clones per phenotype is recommended in order to identify the highest secretor (Zheng et al. 2013). This is an extremely time-consuming aspect of using *P. pastoris*, which otherwise has the potential to be a favourable recombinant expression host, particularly after the creation of a strain with a humanized glycosylation pattern (Hamilton and Gerngross 2007). Similarly to mammalian cells, much of the clonal variation has been attributed to the presence of multiple gene copies or varying integration sites (Aw and Polizzi 2013; Clare et al. 1998; Schwarzhans et al. 2016). However, it is clear that other host-related determinants are also operational. To date, two papers have carried out the most comprehensive investigations into clonal variation, the first by Viader-Salvadό et al. (2006) used amplified fragment length polymorphism (ALFP). By analysing 14 transformants and three control strains, they determined that variation predominantly arose from the transformation process, and that the clones that most closely resembled the host strains gave higher yields. Schwarzhans et al. (2016) also used genomic analysis to investigate clonal variation, yet differences in copy number and integration sites meant that their definition of clonal variation differs from what we have outlined as being genetically identical clones. Their findings indicated that integration loci, copy number and vector orientation were the most fundamental causes of differences to yields.

A greater understanding of clonal variation could simplify the process of selecting clones with a high level of secretion of recombinant proteins. If specific markers or attributes can identify high-copy clones, then it will be possible to use these to look for high-secreting clones, through the use of RT-qPCR. Alternatively, if a particular attribute is found to contribute to higher secretors, then genetic engineering strains to alter these may improve overall productivity. Selecting the correct strain is fundamental for production and manufacturing strategies, as a good secretor can reduce the need for optimisation of media and growth conditions (Hemmerich et al. 2014; Sreekrishna et al. 1997). In order to evaluate the prevalence and underlying causes of clonal variation, nine strains were selected, each transformed with a single copy of the human serum albumin (*HSA*) gene. All strains were subjected to a wide-ranging evaluation to understand the implications of this phenomenon.

## Materials and methods

### Strains and vectors

*Escherichia coli* DH5α (New England Bioline, London, UK) was used for subcloning. *K. phaffii*, more commonly known as *P. pastoris*, GS115 (*HIS4* ^-^), X33 and the pPICZα and pPICZα + *HSA* control strain were obtained from Invitrogen, Carlsbad, CA. The *HSA* gene was amplified using primers CCC GAA TTC ATG AAG TGG GTA ACC TTT ATT TCC C and CCC GAA TTC AAAA ATG AAG TGG GTA ACC TTT ATT TCC C to remove the native signal peptide and add the restriction sites for *Eco*RI and *Not*I, respectively (Fermentas, York, UK). The amplified *HSA* gene was transformed into the vector pPICZα A to generate the pPICZα-*HSA* strains.

### Media

Buffered glycerol-complex medium (BMGY) and buffered methanol-complex medium (BMMY) medium contained 1% (*w*/*v*) yeast extract (Merck, Nottingham, UK), 2% (*w*/*v*) casein peptone (Merck, Nottingham, UK), 100 mM potassium phosphate, pH 6.0, 1.34% (*w*/*v*) yeast nitrogen base (YNB), 4 × 10^−5^% (*w*/*v*) D-biotin, 1% (*v*/*v*) glycerol or 0.5% (*v*/*v*) methanol, respectively. Yeast extract peptone medium (YPD) contained 1% (*w*/*v*) yeast extract (Merck, Nottingham, UK), 2% (*w*/*v*) casein peptone (Merck, Nottingham, UK), 2% (*w*/*v*) dextrose (glucose). For agar plates, 2% (*w*/*v*) agar was included. For selection on Zeocin, all media were supplemented with 100 μg mL^−1^ Zeocin.

### Transformation

Chemically competent DH5α cells were transformed according to the manufacturer’s protocol. *P. pastoris* strains were transformed as suggested by Invitrogen, Carlsbad, CA. Briefly, 100 mL of YPD was inoculated from an overnight culture and left to grow until OD_600_ 1.3–1.5 cells were washed with water and sorbitol prior to resuspension in 1 M ice-cold sorbitol. A 5–10 μg of pPICZα-*HSA* digested with *Pme*I for linearization was inoculated with 80 μL of the ice-cold cells. Cells were pulsed at 2000 V, 25 μF and 200 Ω, for approximately 5 ms, using the GenePulser electroporator in 2-mm cuvettes (Bio-Rad, Hemel Hempstead, UK). Immediately, 1 mL of ice-cold 1 M sorbitol was added to the cuvette and the contents transferred to a sterile Eppendorf tube. The transformation was incubated at 30 °C without shaking for 1–2 h, after which the transformation was plated onto YPD plates containing Zeocin. The plates were incubated for 3–5 days at 30 °C. When selecting clones for analysis, single copies were first streaked onto non-selection YPD plates, left to grow for 3–5 days prior to being restreaked onto YPD Zeocin plates.

### Sequencing

All sequencing reactions were carried out by Eurofins MWG Operon (Ebersberg, Germany).

### Growth conditions

Cultures were grown in 250-mL shake flasks or 24-well microtiter plates for 24 h, at 250 rpm, in the glycerol-containing medium, BMGY. OD_600_ was measured and cultures normalised to OD_600_ 10. The entire culture was centrifuged for 5 min at 4000 rpm at room temperature, the supernatant discarded and the cells resuspended in methanol-containing BMMY and left to grow for a further 24 h. Measurements of HSA secretion were the mean of three biological replicates. The final OD_600_ for each culture was around 20 suggesting that they had all grown at similar rates.

### Titer analysis

HSA titer was determined using the albumin blue fluorescence assay according to the manufacturer’s instructions (Active Motif, La Hulpe, Belgium). Fluoresence was measured at 560 nm, emission 620 nm using Synergy HT multi-detection microplate reader (Bio-Tek, Potton, UK). All samples were measured in triplicate.

### Quantitative PCR (qPCR)

For reverse transcription (RT)-qPCR, RNA was isolated using RiboPure—Yeast Kit according to manufacturer’s instructions (Applied Biosystems, Warrington, UK). Typically, 3 × 10^8^ cells were collected, equivalent to 1 mL culture of OD_600_ 10. Complementary DNA (cDNA) was prepared using the High-Capacity cDNA Archive Kit (Applied Biosystems, Warrington, UK). 1 μg RNA was used in a total reaction volume of 20 μL. RT-qPCR reactions were set up using the 2X SYBR® Green JumpStart Taq Ready Mix (Sigma-Aldrich, Dorset, UK). A Chromo4™ Real-Time Detector using the thermal cycler software Opticon 3 (Bio-Rad, Hemel Hempstead, UK) was used. Data was analysed using the Pfaffl method, based on ΔΔCt (Livak and Schmittgen 2001; Pfaffl 2001) and normalised to *ACT1* as the housekeeping gene. Primers for *ACT1* were GCT TTG TTC CAC CCA TCT GT and TGC ATA CGC TCA GCA ATA CC. Primers for *HSA* were GGT GTT GAT TGC CTT TGC TCA G and GCA TTC ATT TCT CTC AGG TTC TTG. Primers for *HAC1* were CGA CTA CAT TAC TAC AGC TCC ATC A and TGC TGT AAT GTG TAA AGA TGA ATC C, for *PDI* GCC GTT AAA TTC GGT AAG CA and TCA GCT CGG TCA CAT CTT TG and for *KAR2* TCA AAG ACG CTG GTG TCA AG and TAT GCG ACA GCT TCA TCT GG. For gene copy number determination, genomic DNA was extracted using the DNeasy Plant Mini Kit according to the manufacturer’s protocol (Qiagen, Crawley, UK). All other reaction conditions were the same as with RT-qPCR.

### Flow cytometry analysis

After 24 h expression in BMMY, 1 mL of culture was collected through centrifugation and washed in 1x TBS (0.605% Tris, 0.876% NaCl, pH adjusted with 1 M HCl). Samples were washed for a second time before being resuspended in a final volume of 10 mL of buffer to create a 1 in 10 dilution. Samples were stained according to the LIVE/DEAD® FungalLight™ yeast viability kit protocol (Invitrogen Corporation, Paisley, UK). Cells were measured using a FACscan flow cytometer (Becton Dickinson, Oxford, UK). Data was acquired using CellQuest software (Becton Dickinson, Oxford, UK) with samples measured on a high flow rate for 30 s. Data was analysed using Cylogic software (CyFlo Ltd., Turku, Finland).

### Transcriptomic analysis

An Agilent (Agilent Technologies UK Ltd., Wokingham, UK) 8 × 60,000 probe custom microarray was designed by Oxford Genome Technologies (OGT, Oxford, UK) based on the Integrated Genomics *P. pastoris* GS115 genome which contained 5195 open reading frames (ORFs). Analysis was carried out at the Bacterial Microarray Group at St George’s Hospital, London, UK. Cy3-labelled complementary RNA (cRNA) was prepared from 1 μg total RNA using the Agilent One-Colour Quick Amp Labelling Kit according to the manufacturer’s instructions (Agilent Technologies UK Ltd., Wokingham, UK). One-Colour Spike-In controls were labelled together with the RNA samples. Purified samples were hybridised to an Agilent 8 × 60,000 format Sureprint G3 gene expression custom array and incubated overnight in a rotating oven (Agilent Technologies UK Ltd., Wokingham, UK) at 65 °C, 20 rpm. After hybridization, slides were washed for 1 min at room temperature in GE wash buffer 1 and 1 min at 37 °C in GE wash buffer 2 (Agilent Technologies UK Ltd., Wokingham, UK), placed beneath an Ozone Barrier Slide cover (Agilent Technologies UK Ltd., Wokingham, UK) and scanned immediately, using an Agilent High-Resolution Microarray Scanner, at 2 μm resolution. Scanned images were quantified using Feature Extraction software v 10.7.3.1 (Agilent Technologies UK Ltd., Wokingham, UK). Unlike some studies, we did not apply an artificial “fold increase” cutoff at this stage on the grounds that even relatively small changes in regulation of expression of genes such as transcription factors can result in significant global responses (Tarca et al. 2006). Furthermore, the linking of regulation patterns through pathway analysis provides greater robustness than when looking at single genes in isolation.

### Gene expression analysis

Analysis was carried out by Bioinformatics Support Service, Imperial College London, using the Bioconductor package in the R programming language to identify differentially expressed genes (Gentleman et al. 2005). Each of the clonal variation (CV) strains was contrasted to wild-type X33 in order to determine statistically significant differences. The empirical Bayes method was applied to identify statistical significance in contrast between gene expression profiles (Smyth 2004). The false discovery rate (fdr) based on Benjamini and Hochberg’s method, which assumes that all genes are statistically different from one another, was set to be less than 5% (Smyth 2005). Gene functionality was assigned with reference to a created *P. pastoris* genome (www.blugen.org/gbrowse-bin/gbrowse/Pichia/). All microarray data files have been deposited into ArrayExpress (accession code A-MTAB-602).

### Pathway analysis

Pathway analysis was used to identify pathways which were significantly upregulated or downregulated in accordance with the gene expression data. Initially, significantly upregulated or downregulated genes were run through KOBAS (KEGG Orthology Based Annotation System), which assigns genes to pathways based on the KEGG maps specifically for *P. pastoris* (Mao et al. 2005; Wu et al. 2006; Xie et al. 2011). Once pathways were identified the KEGG Search & Colour pathway was used to visually map the differentially expressed genes (Kanehisa and Goto 2000; Kanehisa et al. 2012).

## Results

### Differences in messenger RNA (mRNA) transcript levels variation and UPR upregulation are insufficient to explain clonal variation

**Fig. 1:**
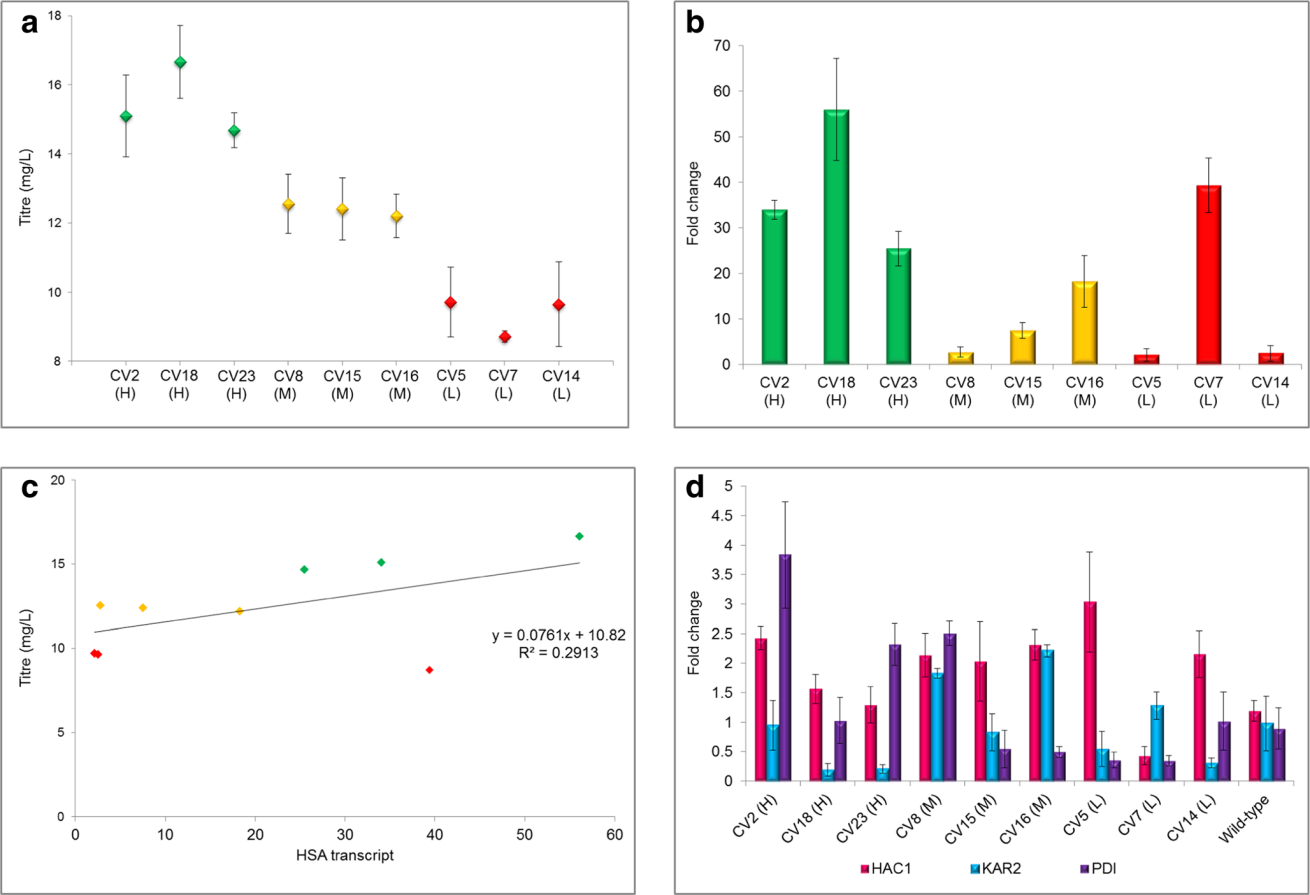
Correlation of secreted HSA titer with *HSA* and UPR-related transcript levels. Clones were expressed in triplicate for 24 h in methanol-containing medium in 250-mL baffled flasks. *Green*—high secretors, *yellow*—mid secretors and *red*—low secretors. **a** Secreted HSA titer levels of single copy *HSA* clones; error bars displayed indicate 95% confidence interval. **b** *HSA* transcript levels of single-copy *HSA* clones calculated by the ΔΔCt method in comparison to *ACT1* as the housekeeping gene. **c** Correlation of *HSA* transcript level (fold change) with HSA titer (mg L^−1^). **d** Transcript levels of *HAC1*, *KAR2* and *PDI* for each of the single-copy *HSA* clones calculated from transcriptomic analysis

To determine the prevalence of clonal variation, 24 transformants were selected from a single transformation of pPICzα-*HSA* into *P. pastoris* GS115 and selected on YPD plates containing 100 μg mL^−1^ Zeocin. HSA titer was calculated after 24 h growth in microtiter plates in methanol-containing medium using the albumin blue fluorescence assay and ranged from 5 to 22.5 mg L^−1^ (data not shown) with a high degree of reproducibility in biological replicates (Fig. 1a). Each clone was then analysed by qPCR and shown to contain a single copy of the *HSA* gene, and the integration site was sequenced and found to be identical, with consistent and faithful recombination into the *AOX1* site in all cases (Supplementary Fig. S1). Although the secretion level of the 24 transformants did not show any discrete grouping, to limit the number of analyses three high secretors, three mid secretors and three low secretors were selected and each grown in a 250-mL baffled flask for 24 h in methanol-containing medium. Whilst the inoculum was standardised, no consideration was given to differences in growth rate or any other contributing factor that may have impacted the titer. The three groups were determined to be statistically independent sets based on HSA titer (Fig. 1a). In the following analysis, high-secreting strains are identified by (H), mid-secreting strains by (M) and low-secreting strains by (L) for ease and convenience.

In order to explore the underlying factors determining the variation in titer, likely indicators, including *HSA* transcript levels, upregulation of components of the unfolded protein response (UPR) and variation in growth rates/cell viability were investigated to determine whether these might have an effect. *HSA* transcript levels were determined by RT-qPCR for each of the nine strains. Despite being statistically distinct groups based on titer (Fig. 1a), transcript levels did not correlate strongly to titer levels (Fig. 1c), except with the removal of CV7(L) as an outlier. Once this had been done, there was a broad correlation between secreted protein titer and transcript level, but transcript levels varied widely within each group. CV7(L), a low secretor, showed transcript levels higher than two of the high-secreting strains and higher than all of the mid secretors (Fig. 1c).

The disconnection between transcript levels and secreted protein titers in this strain points to either a reduced level of translation or a blockage in the secretion pathway. However *HAC1*, *KAR2* and *PDI1* expression levels (Fig. 1d) were not significantly higher than in wild-type (indeed *HAC1* and *PDI1* were lower), so low protein expression cannot be explained by protein degradation via the ER-associated degradation (ERAD) pathway, which suggests that the low titers in this strain potentially result from translational problems. This is analysed in more detail below, providing justification for treatment as a special case. The variation in mid secretors also implies that the problem of clonal variation cannot be explained solely by transcript levels, as CV8(M) shows transcript levels equivalent to the two low secretors, CV5(L) and CV14(L), but secreted HSA titer was significantly higher (Fig. 1c). However, there is a less compelling case for treating this as an outlier. Comparison of the levels of secretion stress, indicated by upregulation of *HAC1*, *KAR2* and *PDI* between the H, M and L groups, did not show any systematic variation which may have explained the different titers observed (Fig. 1d), although *HAC1* expression was higher in all clones except CV7(L). However, unless a variant had arisen with an exceptionally sensitive UPR response, the lack of involvement of UPR in clonal variation of titers derived from a single copy of *HSA* is not surprising; in other studies, we and others (Marx et al. 2009) have observed that it typically takes expression of at least five copies of the *HSA* gene to induce UPR (data not shown).

### Increased cell death is evident in high secretors

**Fig. 2:**
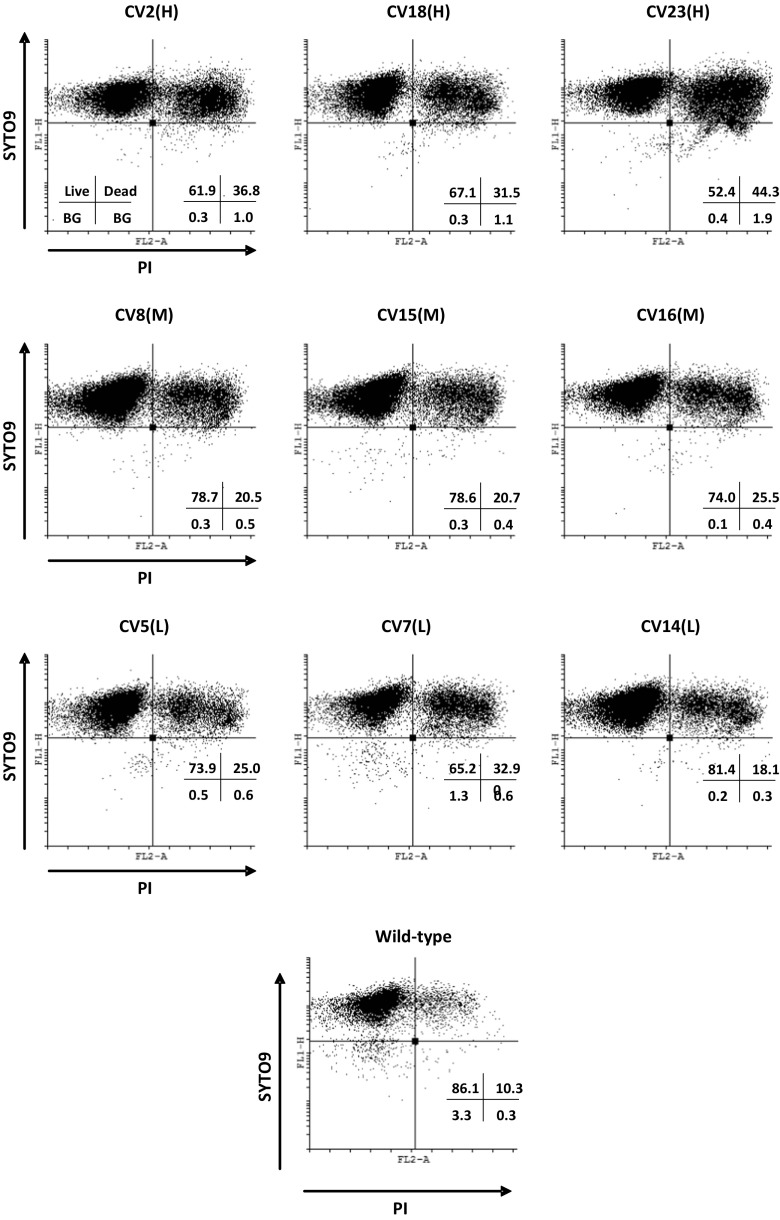
FACS analysis of clonal variants grown for 24 h in methanol-containing medium. FL1-H: SYTO9® a green fluorescent nucleic acid stain indicating live cells, FL2-A: propidium iodide a red fluorescent stain indicating dead cells with a damaged membrane. *BG* background noise. Quadrant displays proportion of live/dead cells and background noise

Growth rates, as determined by the rate of increase in cell density (OD_600_), were compared between high, mid and low secretors, but no significant variation was observed in either a glycerol-based or in the initial adaptation to a methanol-based medium over 8 h (Supplementary Fig. S2). However, as OD_600_ measurements do not differentiate between increased cell size and increased cell numbers nor the proportion of live cells, a live/dead cell flow cytometric assay was performed, analysing the viability of cells after growth in a glycerol-based medium (Supplementary Fig. S3) and a methanol-based medium (Fig. 2). Based on staining with both SYTO9® and PI, there was no correlation between HSA titer and the proportion of live/dead cells with cells grown on glycerol medium, with both the low and high secretors showing a similar proportion of dead cells (Supplementary Fig. S3). However, CV2(H) was anomalous in this regard, showing a higher proportion of dead cells than any of the other strains, including wild-type.

When grown with methanol as the sole carbon source, the high secretors showed an increased proportion of dead cells compared to the other strains, with the exception of CV7(L), which as discussed above, may be under some form of translational stress. CV23(H) exhibited a near 1:1 ratio of live/dead cells. When grown on methanol the proportion of dead cells in the wild-type was lower than any of the other strains, similar to levels initially seen when grown on glycerol, highlighting the effects of the additional burden that protein secretion has on the cells. The absence of any major UPR upregulation suggests that the greater proportion of cell death in the high secretors resulted from a different stress, possibly reflecting the biosynthetic demand of higher levels of protein production. Interestingly, in a cell size comparison by flow cytometry, CV5(L), CV7(L) and CV14(L) were shown to have the largest cells (data not shown).

### Transcriptomic analysis reveals limited correlation between titer and gene expression

In order to further understand the nature of the variation between the strains, transcriptomic analysis was carried out on the nine strains. Strains were grown, in triplicate, for 24 h in glycerol medium, before being induced in methanol medium for 24 h to allow for significant protein production. Variations in expression compared to wild-type with a false discovery rate (fdr) ≤ 0.05 were used to identify differentially expressed genes (Supplementary Fig. S4).

**Fig. 3:**
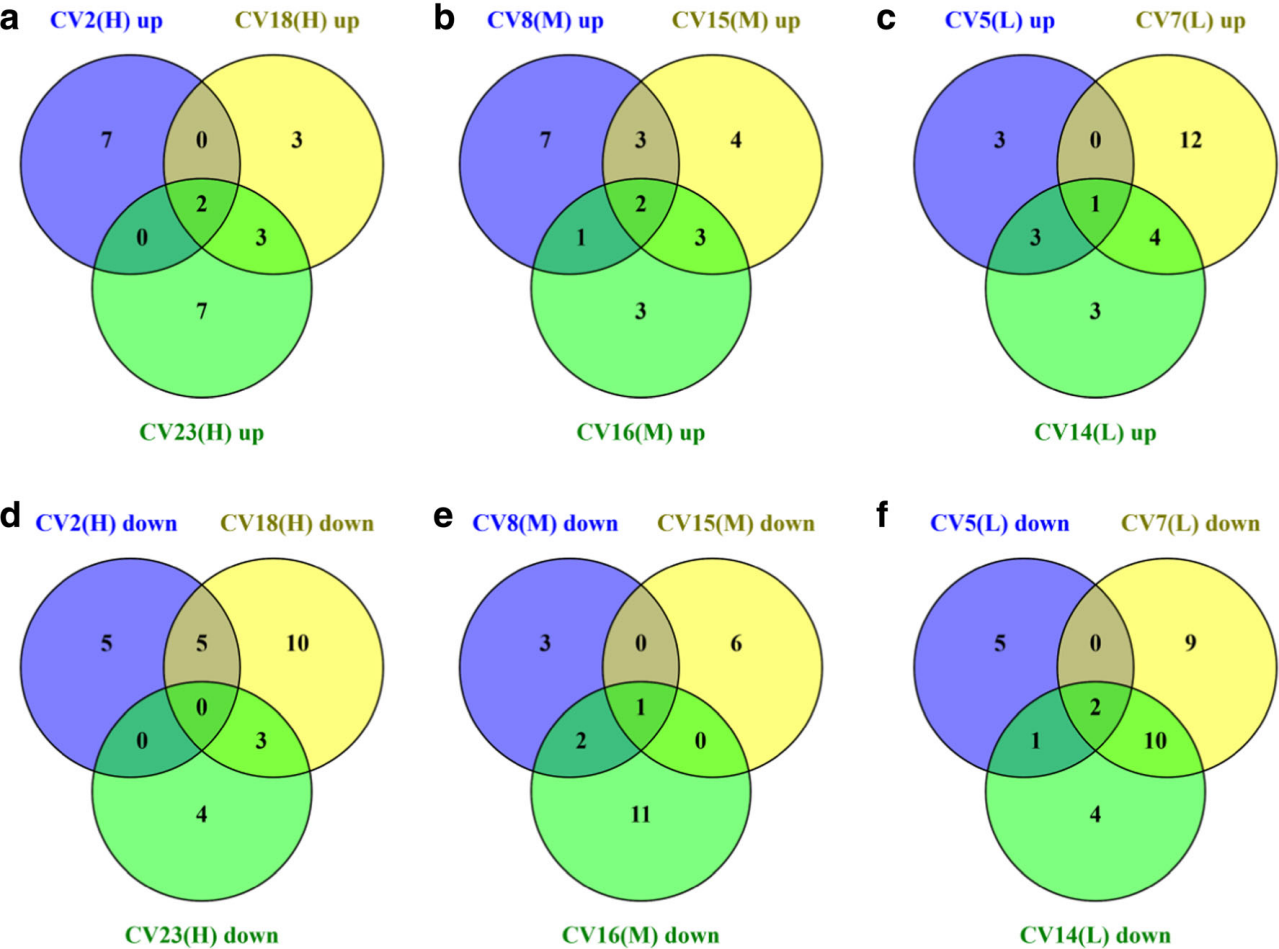
Venn diagrams of upregulated or downregulated pathways in the H, M and L clones. The Venn diagrams indicate the number of KEGG-classified pathways that were significantly upregulated or downregulated in the H, M and L clones, determined by KOBAS pathway analysis. **a** Upregulated in high secretors. **b** Upregulated in mid secretors. **c** Upregulated in low secretors. **d** Downregulated in high secretors. **e** Downregulated in mid secretors. **f** Downregulated in low secretors

To gain an overview of how each of the strains had changed after 24 h of protein expression, significantly upregulated and downregulated genes were analysed using the programme KOBAS to assign differentially expressed genes to KEGG pathways (Wu et al. 2006; Xie et al. 2011). The programme quantitatively determines the significant patterns of whole pathway upregulation or downregulation based on the proportion of genes within that pathway which are upregulated or downregulated, with a threshold of *p* ≤ 0.1 (Fig. 3 and Supplementary Tables S1 and S2).

**Fig. 4:**
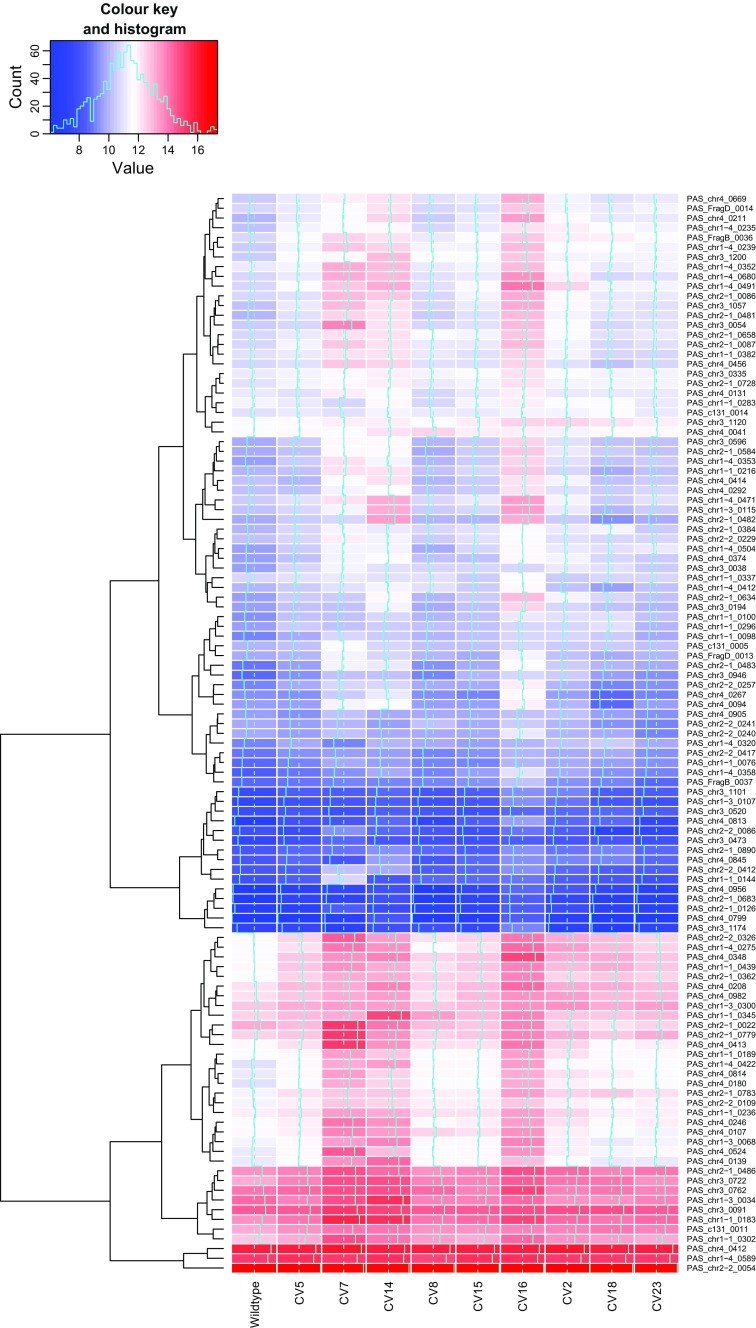
Expression levels of genes-encoding proteins involved in ribosomal proteins. Heat maps of log 2 normalised expression levels of genes-encoding ribosomal proteins, as defined by KEGG families, compared to those of wild-type after 24 h induction with methanol. The associated trees cluster genes with similar expression profiles across all conditions

As expected CV7(L), the strain which has already been highlighted as suffering some sort of translational stress had the highest number of KEGG-classified pathways (17) significantly upregulated and (21) downregulated compared to the wild-type, and this strain is dealt with separately. However, analysis of groups of commonly regulated pathways (Fig. 3) showed that there were very few that were similarly upregulated or downregulated within all members of the titer groups. High secretors only showed two pathways which were significantly upregulated and none that were downregulated in all clones. One of the commonly upregulated pathways was oxidative phosphorylation, and it is clear from the heat maps (Supplementary Fig. S5) that a similar pattern was present in CV16(M) and to a lesser extent CV15(M), but CV8(M), which has already been highlighted as having a low transcript level, was virtually identical to wild-type. Increased oxidative phosphorylation activity is likely to be a consequence of the increased metabolic demand for recombinant protein biosynthesis, rather than an underlying cause. Interestingly, ribosome biogenesis, which typically correlates with growth rate and heterologous protein production, was the other commonly upregulated pathway. However, this was also observed in the medium and low titer groups with the exception of CV7(L), suggesting that heterologous protein expression is able to increase ribosomal biogenesis in a “demand-led” manner. Increased ribosome biogenesis activity requires increased ribosomal protein production to increase ribosome numbers. Of all of the differences in expression between the selected clones, the difference in ribosomal protein expression was the most dramatic, although the extent of these differences did not correlate well with HSA titer (Fig. 4). CV7(L), already highlighted as a special case, CV14(L) and CV 16(M) all showed particularly strong upregulation of expression of a number of genes, many of which were common between all three strains. Therefore, where dramatic changes in ribosomal protein gene expression are observed, these do not correlate directly with high HSA titer, even with the exclusion of CV7(L) from consideration. However, all of the recombinant strains showed some increased expression of ribosomal protein genes, matching the increase in ribosomal biogenesis and consistent with a demand-led increase in protein synthesis in strains expressing heterologous proteins.

In the medium titer group, the only other commonly upregulated pathway was heme biosynthesis; as with oxidative phosphorylation, this would be a consistent response to cells having a higher energy demand for increased protein synthesis. In the low titer group, the commonly upregulated pathway was peroxisome biogenesis, which is surprising as this is a fundamental requirement for methanol oxidation. Whilst a correlation between increased methanol oxidation capacity and increased protein production might have been expected, due to the evident higher energy demand, intuitively we would expect the low titer group to be most similar to the wild-type. However, it is possible that a higher energy demand for other purposes (e.g. general maintenance/repair) in these strains could explain the lower heterologous protein productivity. Removing CV7(L) from the analysis, there were three additional commonly upregulated KEGG groups, namely, ribosome biogenesis (as discussed), purine metabolism and RNA polymerase.

In both CV2(H) and CV18(H), the pathway “protein processing in the ER” was downregulated compared to the wild-type strain. Whilst seemingly counter-intuitive given the higher levels of protein secretion in these strains, this pattern was also observed by Edwards-Jones et al. (2015). Interestingly, a transcriptomic analysis of production of HSA at varying μ showed that higher growth rate correlated to upregulated genes of the secretory pathway, including UPR-related genes (Rebnegger et al. 2014). We saw no differences when monitoring OD_600_ of cells grown in BMGY and for the first 8 h of methanol adaptation (BMMY) between the high, mid and low secretors (Supplementary Fig. S2). The protein processing in ER pathway is also downregulated in the low secretors CV5(L) and CV14(L), so the effect may simply be a response to heterologous protein expression. Given that there is no evidence for significant ER stress in these clones, lower protein processing in the ER probably reflects the greater energy demand in cells producing heterologous proteins resulting in increased peroxisome and lower ER production.

Increased cell lysis can be a consequence of increased ROS, as a consequence of increased hydrogen peroxide formulation either through aerobic metabolism from mitochondria or generated during the metabolism of methanol (Bener Aksam et al. 2008; Zepeda et al. 2014). Upon investigation of transcripts related to ROS, we found no clear trend between the different expressing groups. For instance, *YAP1* (PAS_chr4_0601), which is responsible for the deoxificiation of ROS caused by increased protein folding (Yano et al. 2009), was significantly downregulated in CV7(L), CV14(L), CV15(M), CV16(L) and CV18(H) compared to wild-type, with all other strains showing no significant difference. *TRX1* (PAS_chr4_0284), on the other hand, showed upregulation in the three high-secreting strains and CV16(M), but no significant difference in the other strains compared to wild-type. *NCE103* (PAS_chr4_0578) was upregulated in all of the strains compared to wild-type, whereas for *SOD2* (PAS_chr1-4_0071), only CV18(H) showed significant upregulation to wild-type, with all other strains showing no significant differences. *TSA1* (PAS_chr2-2_0220), which has previously been reported to show strong upregulation under hypoxia conditions (Baumann et al. 2010), showed upregulation in all strains except for CV5(L), CV18(H) and CV23(H). Therefore, from our mixed results, there is no clear consensus regarding the upregulation of genes resulting to ROS; thus, it is impossible to confirm that the increased cell death observed was as a result of an increase in ROS.

**Fig. 5:**
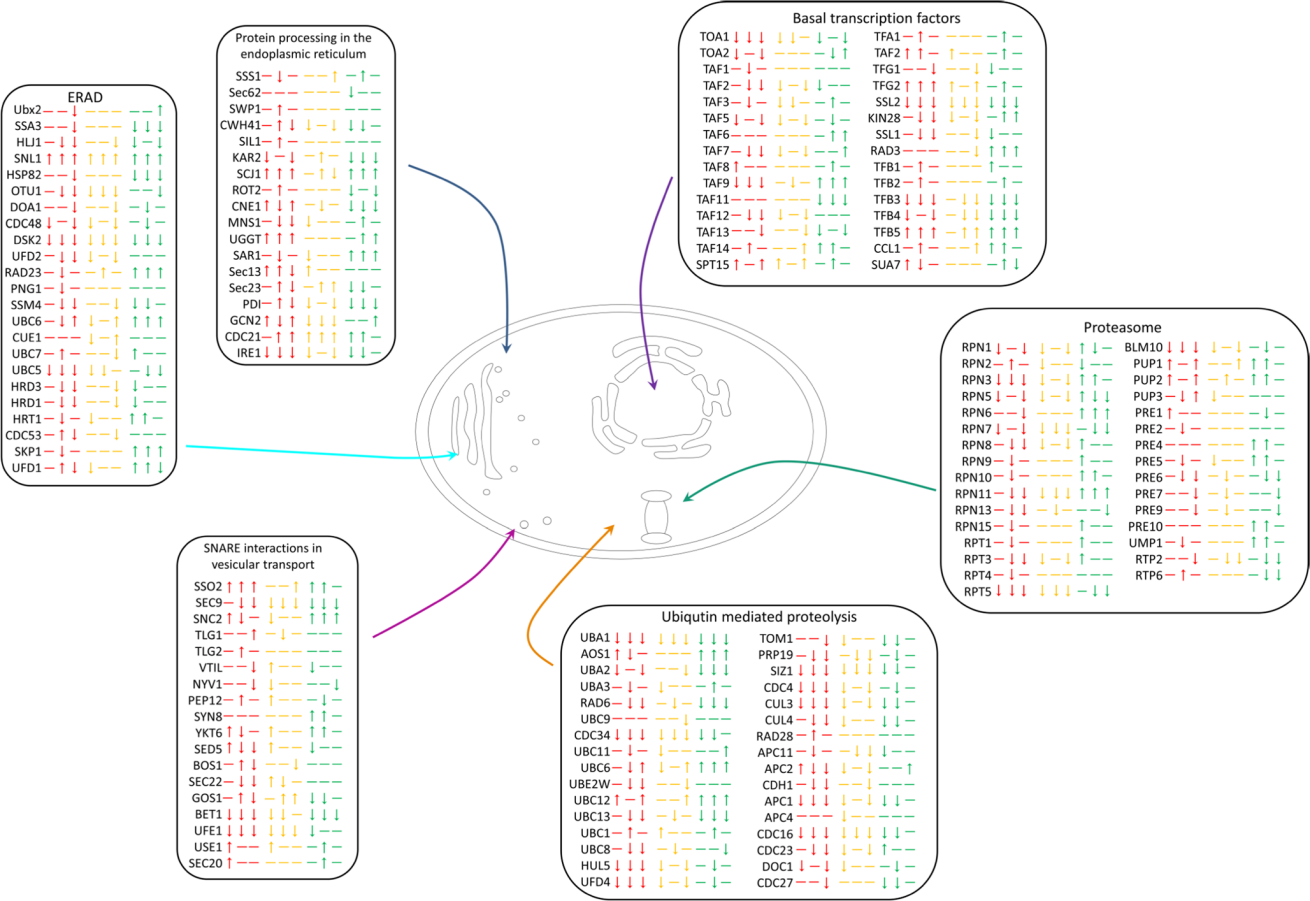
Schematic representation of transcriptomic analysis of steps in the pathways involved in protein production and degradation. Significantly upregulated or downregulated genes (compared to wild-type) are depicted with an *up* or *down arrow*, respectively. A *dash* indicates no significant change compared to wild-type. *Red* indicates low secretors, consistently represented in the order of CV5, CV7 and CV14, *yellow* indicates mid secretors in the order of CV8, CV15 and CV16 and *green* indicates high secretors in the order of CV2, CV18 and CV23

The schematic representation (Fig. 5) shows that whilst no pathways are significantly upregulated or downregulated as a whole according to KOBAS, there are variations that indicate differences between the strains. We have outlined key pathways that are involved in protein production to show how patterns emerge within the pathways that should be taken into consideration. Specifically, there are some individual genes which showed consistent differentiated patterns of expression. For instance *SKP1*, associated with the ERAD pathway, was upregulated in only the high-secreting strains.

On the basis that over 5000 genes have been analysed, it is surprising that so few genes were consistently significantly upregulated or downregulated solely in the high secretors. However, as the boundaries between high, mid and low secretors were set arbitrarily, there may be a continuum of effects. Certainly, there seem to be a number of genes whose expression is upregulated or downregulated solely as a result of heterologous protein expression (particularly when CV7(L) is excluded), and with a well-secreted protein, such as HSA, the metabolic burden associated with the low-high range may not be sufficient to induce dramatic stress responses. It should be noted that, despite the small number of differences evident in the high secretors, there were no genes that are commonly upregulated or downregulated exclusively in the low-secreting strains that were not similarly upregulated or downregulated in either the mid- or high-secreting strains. This implies that there is no specific defect that characterises low secretors.

### CV7(L) is an anomalous strain that shows evidence of nutrient starvation

**Fig. 6:**
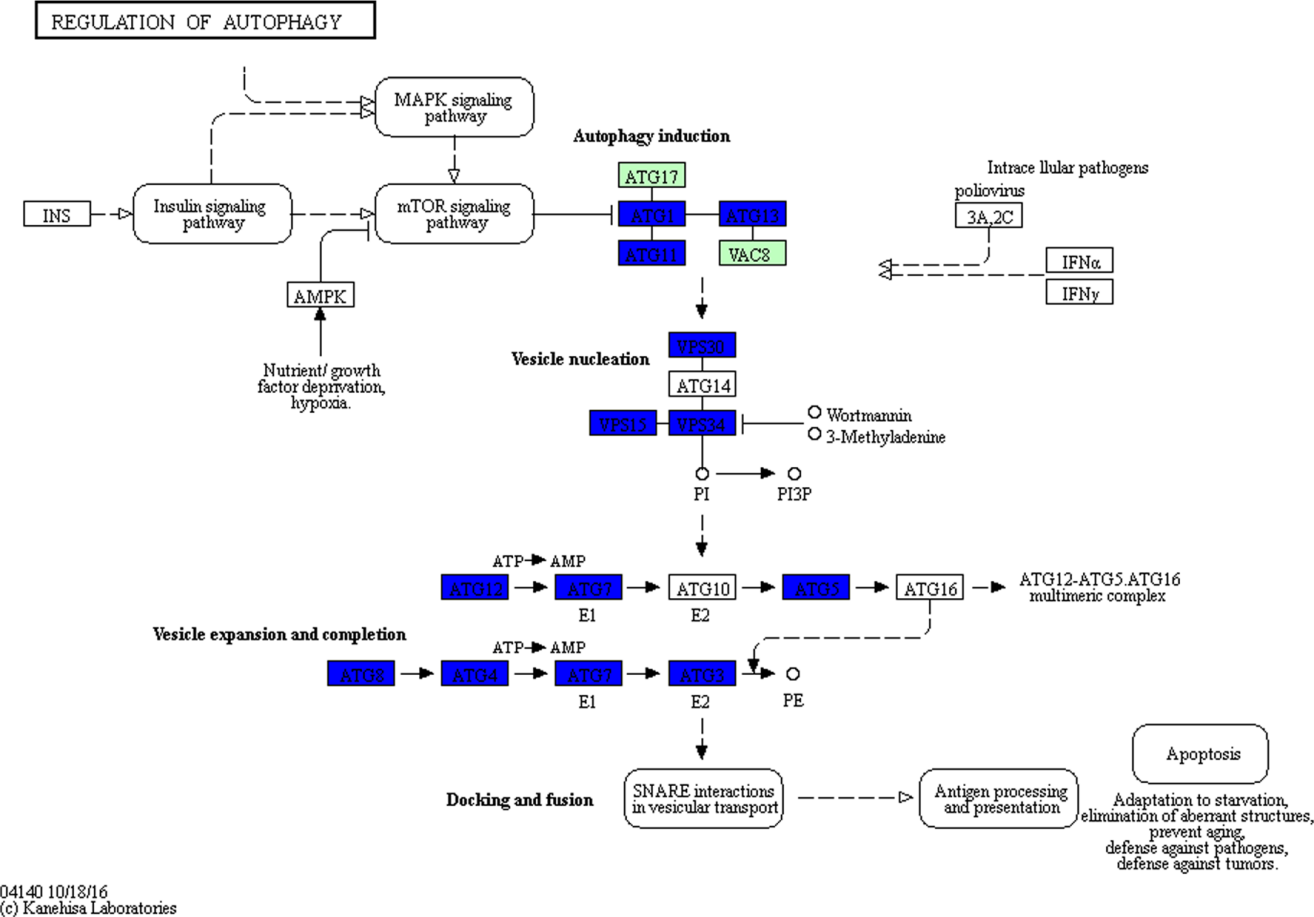
Downregulation of expression of genes in the autophagy pathway of CV7(L) compared to wild-type. Microarray data was mapped using KEGG Mapper Search & Colour pathway. *Green boxes* indicate organism-specific pathways. *Blue boxes* show genes that are significantly downregulated. The figure was generated using the KEGG Mapper Search & Colour pathway programme (Kanehisa and Goto 2000; Kanehisa et al. 2012)

CV7(L) showed different attributes from the other low-secreting strains suggesting that low secretion in this strain was due to a specific defect. Despite the low titer (Fig. 1a), transcript levels of *HSA* were equivalent to the high-secreting strains (Fig. 1b). Furthermore, this strain showed downregulation of expression of almost all of the autophagy pathway genes, with no genes upregulated, which was a unique characteristic of this particular strain (Fig. 6).

All other strains showed consistent upregulation of *ATG7* (data not shown), involved in cytoplasm to vacuole transport and is predicted to be an E1-like activating enzyme essential for the upregulation of autophagy (Meijer et al. 2007; Yuan et al. 1999). Furthermore, the majority of the ubiquitin-mediated proteolysis pathway was downregulated in CV7(L) (Supplementary Fig. S6). Autophagy is required to recycle amino acids for either the production of proteins or for the cellular reorganisation such as production of peroxisomes, which are required during methanol metabolism (Dunn et al. 2005; Nazarko et al. 2011; Vanz et al. 2012). Perhaps the fact that CV7(L) does not upregulate autophagy (more than wild-type) indicates that this basal ability to recycle amino acids is missing. Thus, the cell may be experiencing partial starvation, consistent with the live/dead assay which indicates that after 24 h on methanol 32.9% of the cells were nonviable (Fig. 2).

This strain showed the highest number of genes upregulated or downregulated compared to the other strains with 3394 genes differentially expressed. As a consequence, the number of pathways upregulated and downregulated in CV7(L) was greater than any other strain as determined using KOBAS pathway analysis (Table 1).

**Table 1:** Pathways significantly (*p* < 0.1) upregulated and downregulated in CV7(L) as determined by KOBAS pathway analysis

Pathways upregulated in CV7(L)	Pathways downregulated in CV7(L)
Ribosome	Ubiquitin-mediated proteolysis
Biosynthesis of secondary metabolites	Cell cycle—yeast
Oxidative phosphorylation	MAPK signalling pathway—yeast
Metabolic pathways	Regulation of autophagy
Cysteine and methionine metabolism	Nucleotide excision repair
Lysine biosynthesis	Mismatch repair
Valine, leucine and isoleucine biosynthesis	Meiosis—yeast
Phenylalanine, tyrosine and tryptophan biosynthesis	Proteasome
Alanine, aspartate and glutamate metabolism	DNA replication
Ubiquinone and other terpenoid-quinone biosynthesis	Endocytosis
Terpenoid backbone biosynthesis	Natural killer cell-mediated cytotoxicity
Peroxisome	SNARE interactions in vesicular transport
Sulphur metabolism	Folate biosynthesis
Aminoacyl-tRNA biosynthesis	
mRNA surveillance pathway	
Tyrosine metabolism	
Phenylalanine metabolism	

The majority of the pathways upregulated are involved in de novo amino acid biosynthesis, metabolism and bioenergetics, which supports the theory that this strain is unable to recycle amino acids and is reliant on de novo biogenesis. This clearly affects *HSA* translation, leading to low titers, despite the relative abundance of cognate mRNA.

## Discussion

Clonal variation is such a fundamental aspect of working with *P. pastoris* that it is surprising that, to date, only a few groups have investigated the underlying causes of this phenomenon in detail. Viader-Salvadό et al. (2006) determined that much of the variation arose from the process of transformation; however, they claim that when testing 17 strains (14 transformants and 3 wild-type strains) that 16 different patterns were observed. This implies that there must be some inherent variation in *P. pastoris* as all three control strains resulted in different amplified fragment length polymorphism (AFLP) patterns. Furthermore, the investigation by Schwarzhans et al. (2016) using genome sequencing sets different standards for the identification of clonal variation. As has previously been predicted, much of the variation was as a result of multi-copy clones, vector orientation and loci integration (Aw and Polizzi 2013; Clare et al. 1998; Schwarzhans et al. 2016).

Our in-depth assessment into clonal variation, first and foremost, identifies that differences in titer cannot be solely attributed to integration site or copy number unlike those observed by Schwarzhans et al. (2016). Whilst we did not check whether the method of transformation has an effect on the strains, unlike Viader-Salvadό et al. (2006), electroporation was used rather than spheroplasting, which is deemed more stable (Cregg 2007). Looking at titer and transcript of the heterologous protein alone shows variation occurring that could be attributed to transcriptional blockages (CV8(M)), as well as translational blockages (CV7(L)). This initial investigation highlights that strains differ in secreted protein titer for multiple reasons.

The transcriptomic analysis further highlights the fact that no single attribute is clearly responsible for the presence of high or low secretors. No pathways were either consistently upregulated or downregulated that could be solely attributed to the production of a higher titer of HSA. Additionally, the same can be said for the low secretors, where no pathways were statistically different from the mid and high secretors. However, some individual genes, such as *SKP1*, showed upregulated or downregulated expression in all the high secretors. Future work could utilise these target genes to determine whether these genes are consistently upregulated when other proteins are secreted.

The increase in ribosomes in a demand-led manner contrasts with the current picture of tight control of ribosomal biogenesis based on nutritional status and growth rate, although Santoro et al. (2009) have shown that it is possible to artificially switch on silent ribosomal RNA (rRNA) gene expression in some mammalian cell lines, which increased growth rate and heterologous protein production independently of nutritional status. Lin et al. (2013) saw that in a low-expressing strain, five ribosome biogenesis genes were downregulated compared to nine that were upregulated in a high-expressing strain. They determined that higher expression required higher translation rates for protein synthesis. Furthermore, our finding correlates with research by Graf et al. (2008) who determined that a large number of ribosome biogenesis-related genes were upregulated upon the induction of UPR either through *HAC1* overexpression or when chemically inducing UPR with DTT. The authors noted that this trend was different to what had been previously noted with *Saccharomyces cerevisiae* where upregulation of the UPR through DTT resulted in downregulation of ribosome biogenesis genes (Payne et al. 2008).

As the protein secretion pathway did not show significant upregulation or variation between strains, it was unlikely that specific proteolytic activity association with ER stress (ERAD) would be increased in high secretors. However, general proteolysis associated with proteasomal activity does appear to be activated in the high secretors (Fig. 5), which is significant given the evidence for increased cell lysis of these strains. Whilst both of these effects are likely to be consequences rather than causes of high secretion, increased proteasomal activity in the absence of ER stress could potentially be investigated further to determine its feasibility to be used as a biomarker of high productivity. If key targets regularly show upregulation, irrespective of the protein being produced, then this could be used with RT-qPCR to determine whether this strain will be a high secretor. This will become even more important if experiments into bioreactors determine that similar trends are detectable at both large and small scale, allowing for a more robust analysis of high secretors. Furthermore, identifying potential biomarkers could result in identifying targets for metabolic engineering, which has been the case for *YAP1* overexpression. *YAP1* was initially found to be involved in controlling reactive oxygen species (ROS) (Yano et al. 2009). Delic et al. (2012) determined that increased oxidative protein folding led to changes in the redox state of the cytosol and this had an impact on protein expression. Future work then determined that overexpression of *YAP1* led to a rebalancing of the redox conditions and increased protein yield (Delic et al. 2014).

Fundamentally, the microarray analysis raises the question as to whether clonal variation reflects degrees of impairment of secretion or enhancement. Based on indications from Viader-Salvadό et al. (2006), it would be the former with strains that maintain similarities to the wild-type strain showing the highest titers. This could certainly be argued for CV7(L), which showed the highest variation compared to wild-type. Interestingly, the mid secretors showed the least variation from wild-type, with an average of 2513 genes statistically variable from wild-type compared to 3197 in low secretors and 2944 in high secretors. As observed by Viader-Salvadό et al. (2006), there is clearly some inherent variation within the wild-type strain, which may account for a lot of the clonal variation observed in the transformants. Characterising this will be essential for future studies.

The increased cell death in the high-secreting strains could lend itself to the development of even higher-secreting strains, if cell death could be reduced. This has been investigated by Weis et al. (2004) who limited cell death through a strict feeding strategy. It was ascertained that cell death increased with starvation, which is evident in CV7(L) (Fig. 2) and is known to occur in both shake flasks as well as microtiter plates. Therefore, repeating these experiments in a controlled-feed environment may further differentiate the strains and shed more light on the underlying causes of clonal variation. Our experimental procedure using shake flasks did not allow us to tightly control growth parameters, as would be the case with bioreactors. However, it is important to note that during initial strain selection, most screening is performed on small scale, either using microtiter plates or shake flasks. It would be of fundamental value to investigate whether similar trends are observable using strict feeding regimes using bioreactors. Furthermore, Rebnegger et al. (2014) showed with transcriptomic analysis that a higher μ resulted in upregulation of protein secretion-related proteins, including UPR genes. Therefore, attempts to characterise a feeding programme with the high secretors with an increased μ and an decreased cell death may result in a more pronounced transcriptomic profile showing differences between the high, mid and low secretors (Rebnegger et al. 2014).

Clonal variation in *P. pastoris* is often attributed to differences in copy number and the integration site of the vector. However, our in-depth experiments show that significant variation occurs due to factors other than vector integration. Whilst we were unable to pinpoint precise underlying causes of clonal variation, we have highlighted the prevalence and begun to investigate identifiers that could be utilised in future experiments. This raises the question as to where the major variation arises, whether from intrinsic differences within the host strain population or whether the transformation or selection method can give rise to the variation seen. A study of variation within the host strain could provide a mechanism for preselection of potentially good secretors for transformation, thus minimising the subsequent screening exercise.

## Electronic supplementary material

ESM 1 (PDF 2112 kb)
