# Supplementary material for: Insights into the prevalence and underlying causes of clonal variation through transcriptomic analysis in Pichia pastoris
Source: Appl Microbiol Biotechnol. 2017 May 22;101(12):5045–58. doi: 10.1007/s00253-017-8317-2 (PMC5486821; doi:10.1007/s00253-017-8317-2)
Supplement: Supplementary file 1 — (PDF 2112 kb) [file 253_2017_8317_MOESM1_ESM.pdf]

Applied Microbiology and Biotechnology

Insights into the prevalence and underlying causes of clonal variation through transcriptomic analysis in *Pichia pastoris*

Rochelle Aw[1,2], Geraint R Barton[3] and David J. Leak [1,4*]

[1]Department of Life Sciences, Imperial College London, London, SW7 2AZ

[2]Centre for Synthetic Biology and Innovation, Imperial College London, London, SW7 2AZ

[3]Centre for Integrative Systems Biology and Bioinformatics, Imperial College London, London, SW7 2AZ

[4] Current address: Department of Biology & Biochemistry, University of Bath, Bath BA2 7AY, UK

[*]Corresponding author

Email: d.j.leak@bath.ac.uk

Tel: +44 (0) 1225 384309

Supplementary Materials

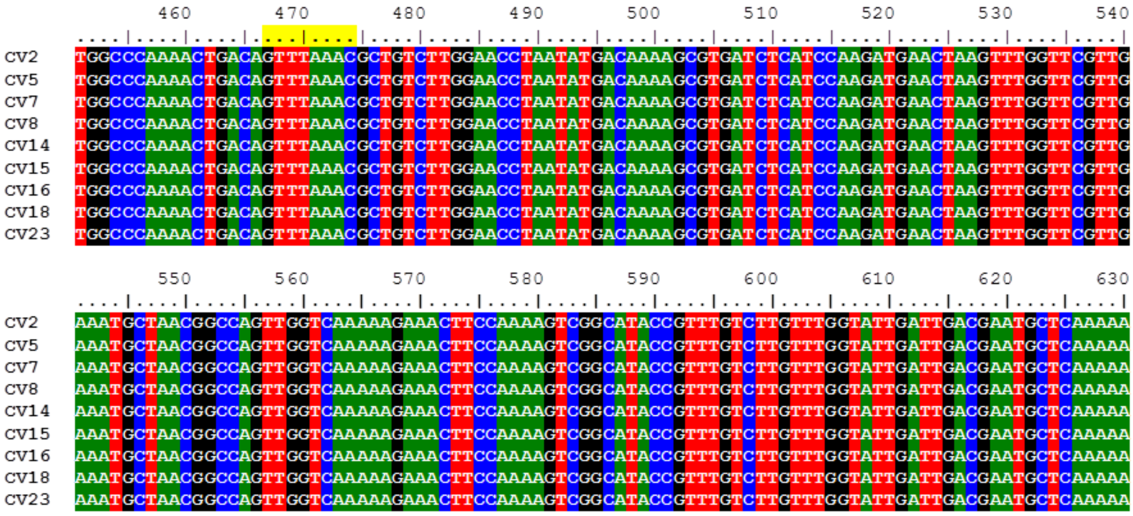

Supplementary Fig. S1 Sequence alignment of the *HSA* integration site for clones used in microarray analysis. Sequence alignment of the region around the *Pme*I directed integration site of clones CV2, CV5, CV7, CV8, CV14, CV15, CV16, CV18 and CV23. *Pme*I recognises and cuts at the sequence GTTTAAAC, located at 467 and highlighted in yellow

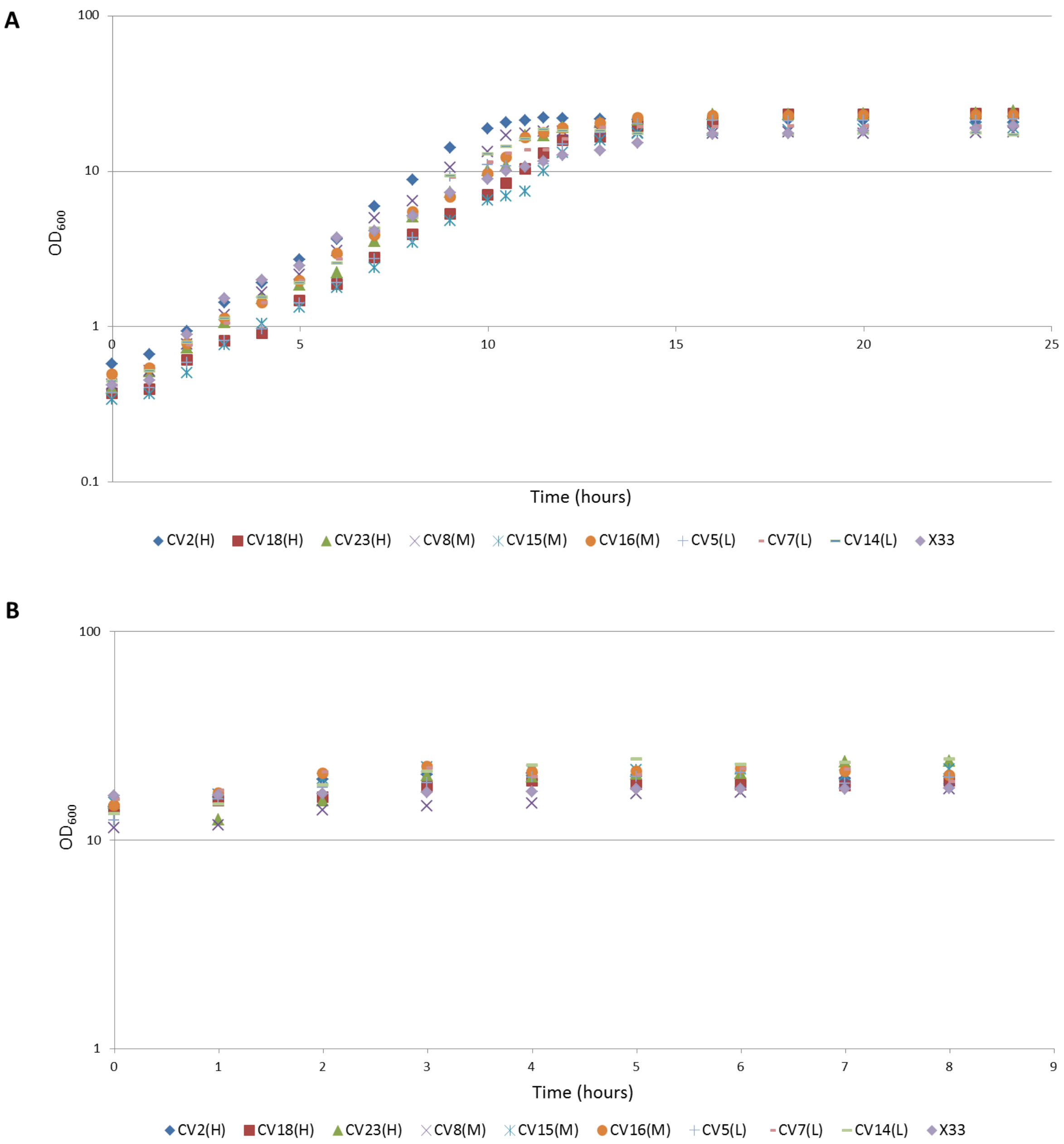

Supplementary Fig. S2 Growth curves of clonal variation strains. Growth curves were monitored by sampling the cultures every hour and the OD_{600} was recorded. Growth in both glycerol and methanol medium was recorded. A) Growth curves in BMGY medium for 25 hours. B) Growth curves in BMMY medium for 8 hours.

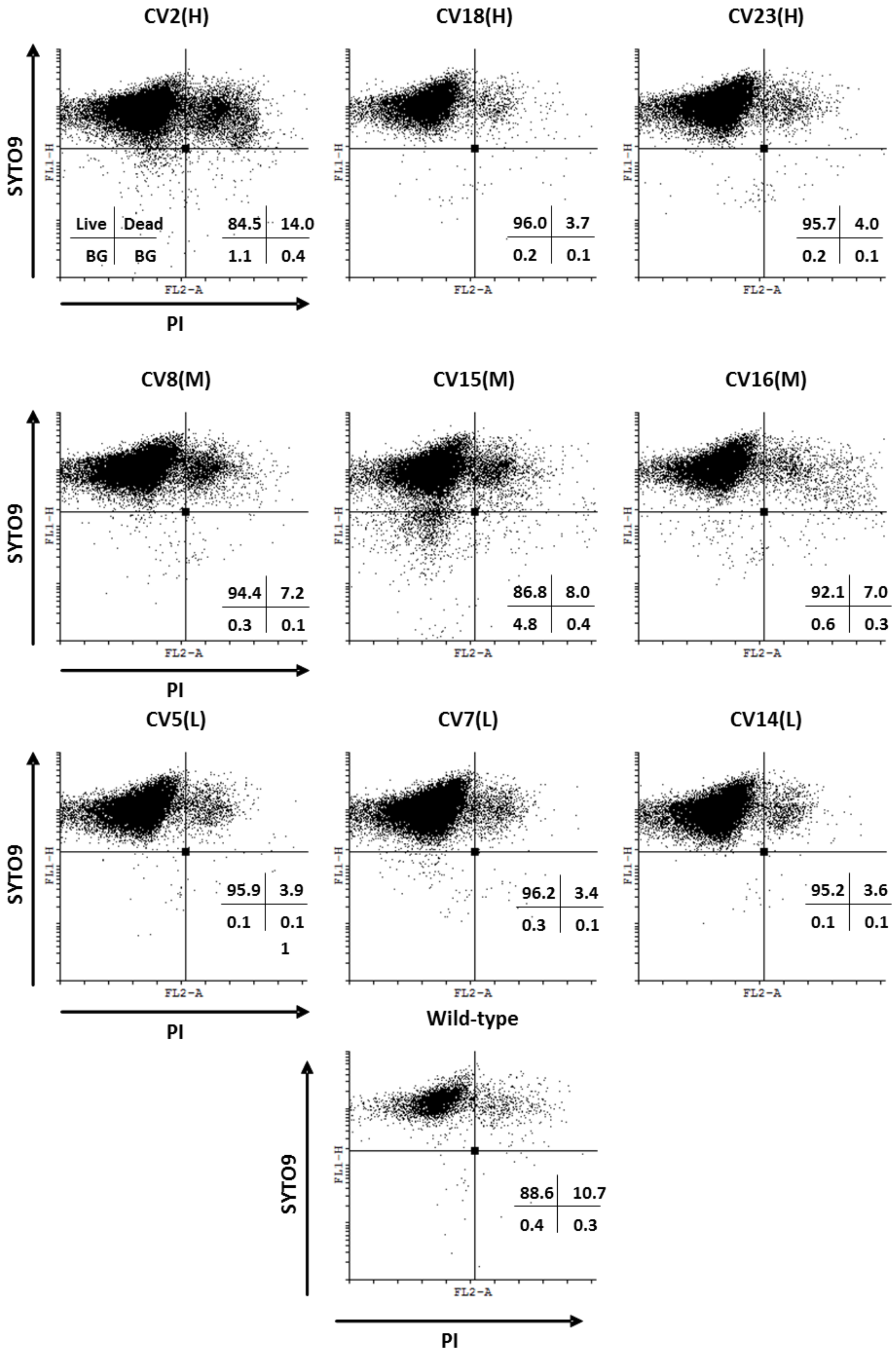

Supplementary Fig. S3 FACS analysis of clonal variants grown for 24 hours in glycerol containing

medium. FL1-H: SYTO9® a green fluorescent nucleic acid stain indicating live cells, FL2-A: propidium iodide a red fluorescent stain indicating dead cells with a damaged membrane. BG: Background noise. Quadrant displays proportion of live/dead cells and background noise.

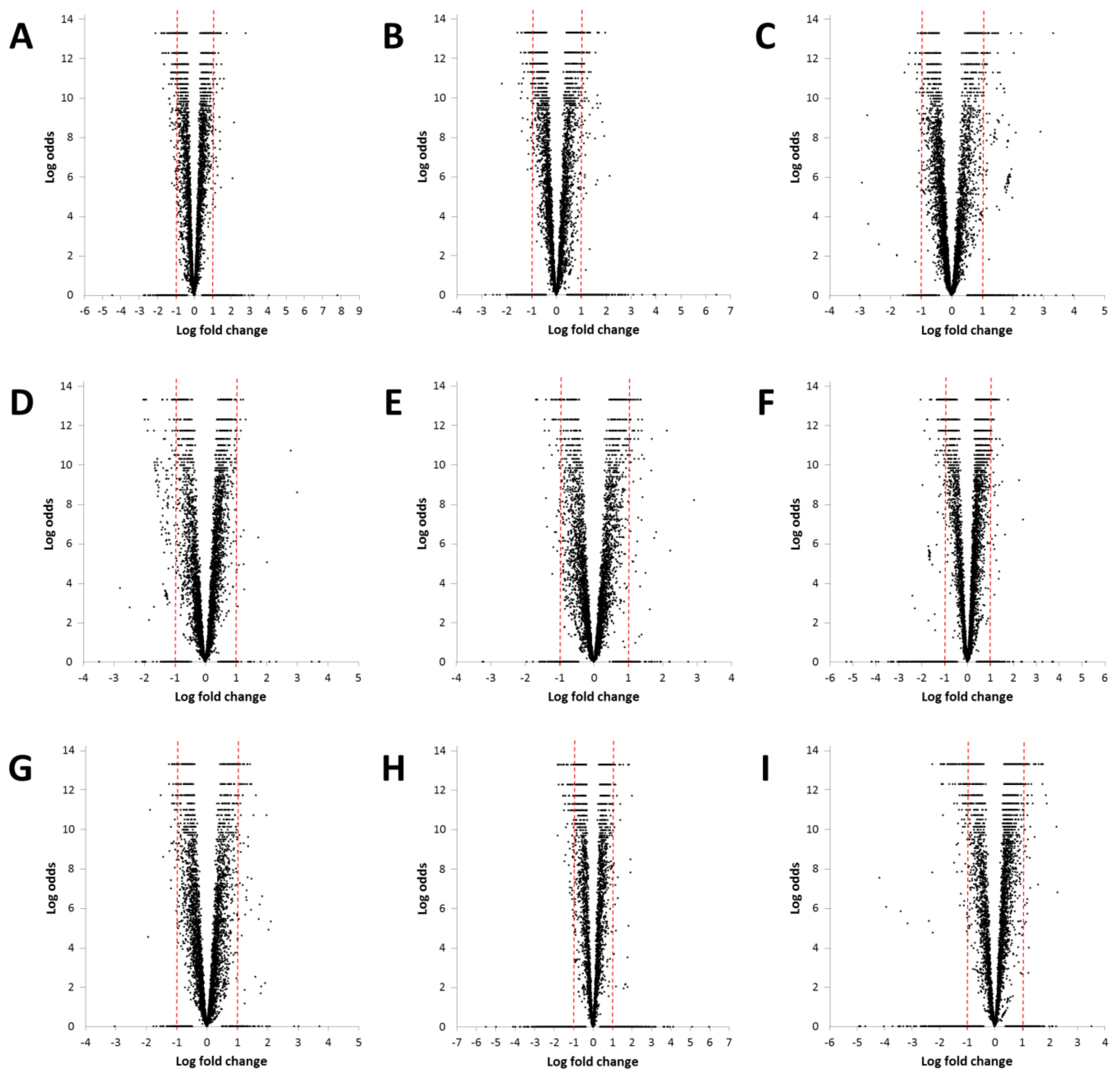

Supplementary Fig. S4 Volcano plots of fold changes vs. adjusted *p* values. A) CV2(H); B) CV18(H); C) CV23(H); D) CV8(M); E) CV15(M); F) CV16(M); G) CV5(L); H) CV7(L); I) CV14(L). Red lines are added for reference to show the boundary of a hypothetical 2 fold change cut-off. No cut-off was used in the analysis presented here, which relied purely on having a false discovery probability of <0.05 (Smyth 2005).

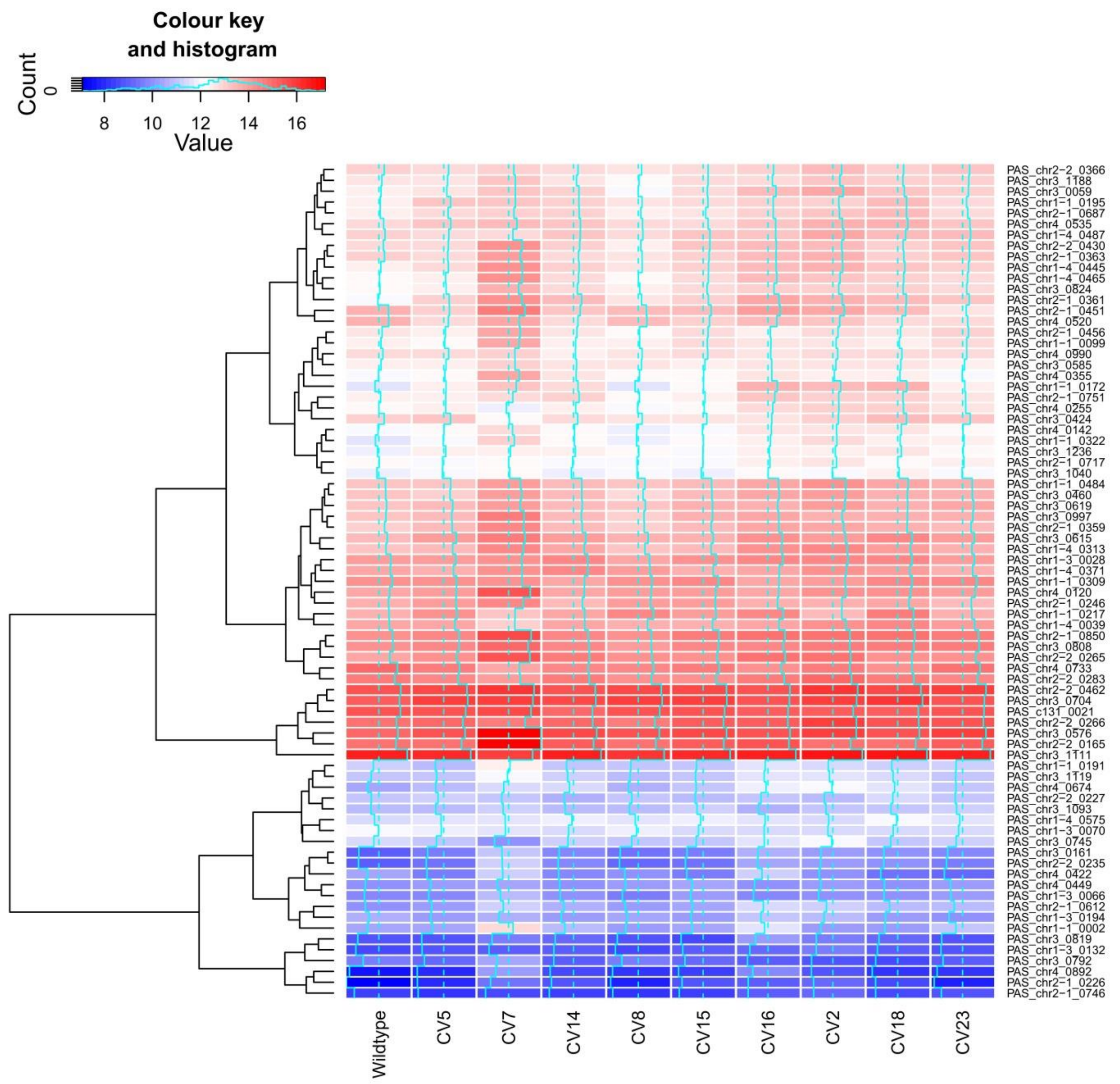

Supplementary Fig. S5 Expression levels of genes encoding proteins involved in oxidative phosphorylation. Heat maps of log 2 normalised expression levels of genes encoding proteins involved in oxidative phosphorylation, defined by KEGG families, compared to those of wild-type after 24 hour induction with methanol. The associated trees cluster genes with similar expression profiles across all conditions

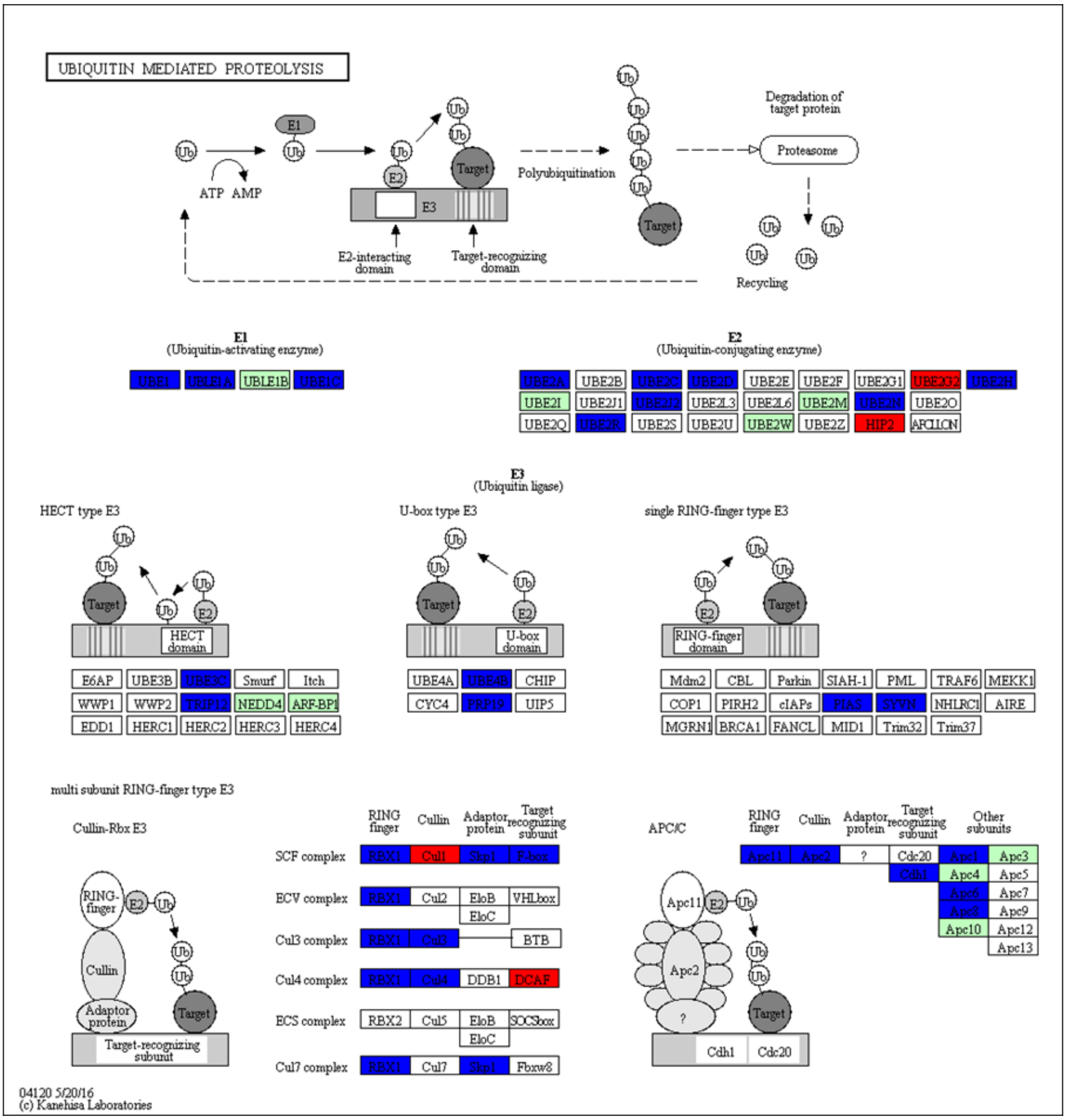

Supplementary Fig. S6 Genes up- or downregulated in the ubiquitin mediated proteolysis pathway in CV7(L) compared to wild-type. Microarray data was mapped using KEGG Mapper Search & Colour pathway. Blue genes indicate downregulation compared to wild-type and red genes indicate upregulation. Green boxes indicate organism-specific pathways. The figure was generated using the KEGG Search&Color Pathway programme (Kanehisa and Goto 2000; Kanehisa et al. 2012).

Supplementary Table S1. Upregulated pathways determined by KOBAS pathway analysis

CV5 (L)	CV7 (L)	CV14 (L)	CV8 (M)	CV15 (M)	CV16 (M)	CV2 (H)	CV18 (H)	CV23 (H)
Fatty acid metabolism	Alanine, aspartate and glutamate metabolism	Arginine and proline metabolism	Alanine, aspartate and glutamate metabolism	ABC transporters	DNA replication	Glycerophospholipid metabolism	Basal transcription factors	Cell cycle - yeast
Peroxisome	Aminoacyl-tRNA biosynthesis	Biosynthesis of secondary metabolites	Arginine and proline metabolism	Arginine and proline metabolism	Oxidative phosphorylation	Nicotinate and nicotinamide metabolism	Biosynthesis of unsaturated fatty acids	Homologous recombination
Purine metabolism	Biosynthesis of secondary metabolites	Cysteine and methionine metabolism	Biosynthesis of secondary metabolites	Biosynthesis of secondary metabolites	Porphyrin and chlorophyll metabolism	Oxidative phosphorylation	Oxidative phosphorylation	Meiosis - yeast
Ribosome biogenesis in eukaryotes	Cysteine and methionine metabolism	Peroxisome	Fatty acid metabolism	Glycolysis / Gluconeogenesis	Purine metabolism	Proteasome	Peroxisome	Mismatch repair
RNA polymerase	Lysine biosynthesis	Purine metabolism	Glycerophospholipid metabolism	Oxidative phosphorylation	Pyrimidine metabolism	Ribosome	Ribosome biogenesis in eukaryotes	Oxidative phosphorylation
Sphingolipid metabolism	Metabolic pathways	Pyrimidine metabolism	Pentose and glucuronate interconversions	Peroxisome	Ribosome	Ribosome biogenesis in eukaryotes	RNA degradation	Peroxisome
Spliceosome	mRNA surveillance pathway	Ribosome	Pentose phosphate pathway	Porphyrin and chlorophyll metabolism	Ribosome biogenesis in eukaryotes	RNA transport	Spliceosome	Phosphatidylinositol signaling system
	Oxidative phosphorylation	Ribosome biogenesis in eukaryotes	Peroxisome	Ribosome	RNA polymerase	Steroid biosynthesis	Sulfur relay system	Ribosome biogenesis in eukaryotes
	Peroxisome	RNA polymerase	Phenylalanine metabolism	Ribosome biogenesis in eukaryotes	RNA transport	Tyrosine metabolism		RNA degradation
	Phenylalanine metabolism	RNA transport	Porphyrin and chlorophyll metabolism	RNA transport				Sphingolipid metabolism
	Phenylalanine, tyrosine and tryptophan biosynthesis	Ubiquinone and other terpenoid-quinone biosynthesis	Purine metabolism	Sulfur metabolism				Spliceosome
	Ribosome		Ribosome biogenesis in eukaryotes	Ubiquinone and other terpenoid-quinone biosynthesis				Ubiquinone and other terpenoid-quinone biosynthesis
	Sulfur metabolism		Spliceosome					
	Terpenoid backbone biosynthesis							
	Tyrosine metabolism							
	Ubiquinone and other terpenoid-quinone biosynthesis							
	Valine, leucine and isoleucine biosynthesis							

Supplementary Table S2 Downregulated pathways determined by KOBAS pathway analysis

CV5 (L)	CV7 (L)	CV14 (L)	CV8 (M)	CV15 (M)	CV16 (M)	CV2 (H)	CV18 (H)	CV23 (H)
Aminoacyl-tRNA biosynthesis	Basal transcription factors	Amino sugar and nucleotide sugar metabolism	Cell cycle - yeast	Basal transcription factors	Aminoacyl-tRNA biosynthesis	Alanine, aspartate and glutamate metabolism	Alanine, aspartate and glutamate metabolism	Basal transcription factors
Fatty acid biosynthesis	Base excision repair	Basal transcription factors	MAPK signaling pathway - yeast	Non-homologous end-joining	Cyanoamino acid metabolism	Cell cycle - yeast	Amino sugar and nucleotide sugar metabolism	Endocytosis
Glutathione metabolism	Cell cycle - yeast	Cell cycle - yeast	Mismatch repair	One carbon pool by folate	Endocytosis	Fatty acid biosynthesis	Aminoacyl-tRNA biosynthesis	Proteasome
Glycine, serine and threonine metabolism	DNA replication	Endocytosis	Protein processing in endoplasmic reticulum	Proteasome	Glycine, serine and threonine metabolism	Lysine biosynthesis	Biosynthesis of secondary metabolites	Protein processing in endoplasmic reticulum
Phenylalanine, tyrosine and tryptophan biosynthesis	Endocytosis	Galactose metabolism	Ubiquitin mediated proteolysis	Riboflavin metabolism	Lysine biosynthesis	MAPK signaling pathway - yeast	Glycine, serine and threonine metabolism	Riboflavin metabolism
Protein processing in endoplasmic reticulum	Folate biosynthesis	Glutathione metabolism	Various types of N-glycan biosynthesis	SNARE interactions in vesicular transport	MAPK signaling pathway - yeast	Methane metabolism	Lysine biosynthesis	SNARE interactions in vesicular transport
Riboflavin metabolism	Homologous recombination	MAPK signaling pathway - yeast		Ubiquitin mediated proteolysis	Mismatch repair	Pentose phosphate pathway	MAPK signaling pathway - yeast	Steroid biosynthesis
Ubiquitin mediated proteolysis	MAPK signaling pathway - yeast	Mismatch repair			Natural killer cell mediated cytotoxicity	Phenylalanine, tyrosine and tryptophan biosynthesis	Natural killer cell mediated cytotoxicity	
	Meiosis - yeast	Natural killer cell mediated cytotoxicity			N-Glycan biosynthesis	Starch and sucrose metabolism	Nitrogen metabolism	
	Mismatch repair	N-Glycan biosynthesis			Nucleotide excision repair	Vitamin B6 metabolism	One carbon pool by folate	
	Natural killer cell mediated cytotoxicity	Non-homologous end-joining			Phenylalanine, tyrosine and tryptophan biosynthesis		Phenylalanine metabolism	
	N-Glycan biosynthesis	Nucleotide excision repair			Regulation of autophagy		Phenylalanine, tyrosine and tryptophan biosynthesis	
	Nucleotide excision repair	Protein processing in endoplasmic reticulum			Starch and sucrose metabolism		Protein processing in endoplasmic reticulum	
	Phosphatidylinositol signaling system	Regulation of autophagy			Ubiquitin mediated proteolysis		Riboflavin metabolism	
	Proteasome	SNARE interactions in vesicular transport					Starch and sucrose metabolism	
	Protein processing in endoplasmic reticulum	Starch and sucrose metabolism					Steroid biosynthesis	
	Regulation of autophagy	Ubiquitin mediated proteolysis					Tyrosine metabolism	
	RNA degradation						Ubiquitin mediated proteolysis	
	SNARE interactions in vesicular transport							
	Ubiquitin mediated proteolysis							
	Various types of N-glycan biosynthesis							
